# Sex-Specific Differences in Extracellular Vesicle Protein Cargo in Synovial Fluid of Patients with Osteoarthritis

**DOI:** 10.3390/life10120337

**Published:** 2020-12-10

**Authors:** Ravindra Kolhe, Virgenal Owens, Ashok Sharma, Tae Jin Lee, Wenbo Zhi, Umar Ghilzai, Ashis K. Mondal, Yutao Liu, Carlos M. Isales, Mark W. Hamrick, Monte Hunter, Sadanand Fulzele

**Affiliations:** 1 Department of Pathology, Augusta University, Augusta, GA 30912, USA; rkolhe@augusta.edu (R.K.); AMONDAL@augusta.edu (A.K.M.); cisales@augusta.edu (C.M.I.); mhamrick@augusta.edu (M.W.H.); 2 Department of Orthopaedic Surgery, Augusta University, Augusta, GA 30912, USA; virgenal.owens@atriumhealth.org (V.O.); UGHILZAI@augusta.edu (U.G.); MOHUNTER@augusta.edu (M.H.); 3 Department of Orthopaedic Surgery, Carolinas Medical Center, Charlotte, NC 28203, USA; 4 Center for Biotechnology and Genomic Medicine, Augusta University, Augusta, GA 30912, USA; ASSHARMA@augusta.edu (A.S.); TALEE@augusta.edu (T.J.L.); WZHI@augusta.edu (W.Z.); 5 Cell Biology and Anatomy, Augusta University, Augusta, GA 30912, USA; YUTLIU@augusta.edu; 6 Department of Medicine, Augusta University, Augusta, GA 30912, USA; 7 Institute of Healthy Aging, Augusta University, Augusta, GA 30912, USA

**Keywords:** gender, cartilage degeneration, exosomes, mass spectrometry

## Abstract

Women are at a significantly higher risk of developing osteoarthritis (OA) compared to males. The pathogenesis of osteoarthritis (OA) in women is poorly understood. Extracellular vesicles (EVs) have been shown to play an essential role in numerous signaling processes during the pathogenesis of age-related diseases via paracrine signaling. Molecular profiling of the synovial fluid-derived EVs cargo in women may help in the discovery of novel biomarkers and therapeutics for the treatment of OA in women. Previously, we reported that synovial fluid-derived EV miRNA cargo differs in a sex-specific manner. This study aims to characterize synovial fluid-derived EV protein cargo in OA patients. Our data showed sex-specific EVs protein content in OA. We found haptoglobin, orosomucoid, and ceruloplasmin significantly up-regulated, whereas apolipoprotein down-regulated in female OA EVs. In males, we discovered β-2-glycoprotein, and complement component 5 proteins significantly up-regulated and Spt-Ada-Gcn5 acetyltransferase (SAGA)-associated factor 29 down-regulated in male OA EVs. Database for Annotation, Visualization, and Integrated Discovery (DAVID) and QuickGO analysis revealed OA-specific protein involvement in several biological, molecular, and cellular pathways, specifically in inflammatory processes. In conclusion, synovial fluid EV protein content is altered in a sex-specific manner with OA, explaining the increased prevalence and severity of OA in women.

## 1. Introduction

Osteoarthritis (OA) of the knee joint is the most common form of arthritis and affects millions of people worldwide. It is the most common cause of disability among adults in the US, affecting approximately 27 million people in the US aged 25 and older, with increased prevalence in older individuals [1]. The prevalence is higher in women than men and increases after menopause [2]. Women often present with more advanced OA, more pain, and increased disability compared to men, with women being three times more likely to have hip or knee replacement surgery [3,4]. In addition, in an increasingly aging population with a higher life expectancy, OA will only become more of a burden for patients. The impact of OA on the lives of patients is pervasive, as it affects functional mobility, leads to increased health care usage and economic burden, and contributes to mortality rates [1]. In conjunction with increasing co-morbid disease states, including diabetes, obesity, and hyperlipidemia, OA will only become a more prevalent disease in the future [5]. Currently, there are no effective treatments for OA, with much of the management being centered on slowing the progression of the disease. The diagnosis of OA primarily consists of physical examination and radiographic findings, which can only be used in diagnosis at later stages of OA, but not for early diagnosis [6]. Early diagnosis is crucial for better and more effective application of conservative and modern therapeutic approaches (stem cell-based) to prevent or delay disease progression [7]. The conservative therapeutic approaches such as physical exercise, vibration, oxygen-ozone therapy, and hyaluronic acid are effective if OA is diagnosed early [8,9,10,11,12]. Much of this inability to effectively diagnose and treat OA is based on the limited knowledge of the disease’s pathogenesis. The synovial fluid of joints has recently become an area of intense focus, as many of the fundamental changes of OA are evident in the synovial fluid [13,14,15]. Synovial fluid is a unique, viscous fluid present in articular joints and surrounded by the synovial membrane and articular chondrocytes at the terminal ends of bones.

Synovial fluid contains various cellular metabolites, including extracellular vesicles (EVs). EVs are approximately 40–100 nm in diameter and are composed of protein, lipid, and small RNA [15,16,17,18,19,20,21]. We previously reported that synovial fluid-derived EVs altered miRNA cargo in a gender-specific manner [15]. The EV protein cargos play an important role in autocrine and paracrine signaling for various metabolic activities during inflammatory processes and disease states [15,16,17,18,19,20,21]. We hypothesized that synovial fluid-derived EV protein cargo might be altered in OA and play a significant role in altering the immune state of the joint, which may have a profound effect on the progression of OA.

This study aimed to identify novel protein cargo carried by EVs in synovial fluid of male and female OA patients. EVs were isolated from the synovial fluid of osteoarthritis and non-osteoarthritic patients, and protein mass spectrometry analysis was performed. We found that EV protein cargo not only differs in the OA population but also in a sex-specific manner. Further bioinformatics analysis showed that these proteins play an essential role in various inflammatory and degenerative diseases.

## 2. Materials and Methods

### 2.1. Patient Samples

The ethical committee (Code: 657441-24) approved all methods, following the relevant guidelines and regulations of Augusta University. Discarded human synovial fluid waste samples used for this study were de-identified and did not require informed consent. The studies were completed with prior approval from the Augusta University Institutional Review Board (IRB). Knee joint synovial fluid from both healthy and osteoarthritic knees was obtained from patients undergoing arthrocentesis/total knee arthroplasty procedures. Donors with severe complications (diabetes, hypertension, HIV, and others) and synovial fluid contaminated with blood were excluded. The synovial fluid was transported to the laboratory and immediately used for exosome isolation after it was obtained from the operating room at the time of surgery.

### 2.2. Preparation of Exosome-Enriched Fractions

A step-wise centrifugation method was used to prepare exosome fractions [15,22,23]. To remove any cell debris, 1 mL of synovial fluid was diluted with 2 mL of phosphate-buffered saline (PBS) and briefly centrifuged at 3000 rpm for 20 min, followed by Total Exosome Isolation Reagent (Life Technologies, Carlsbad, CA, USA) to isolate exosomes as per manufacturer’s protocol. This protocol involved initial precipitation followed by centrifugation. After centrifugation, pellets were dissolved in 200 μL of phosphate-buffered saline (PBS) as exosome-enriched fractions.

### 2.3. Exosome Protein Extraction, Digestion, and LC-MS/MS Analysis

Peptide digestion and mass spectrometry were performed as per our published method [24,25]. Initially, the exosome samples were dried overnight by lyophilization. Furthermore, 100 μL freshly made 50 mM ammonium bicarbonate buffer with 0.1% (w/v) RapiGest SF Surfactant (Waters), and 10 mM dithiothreitol were added into the sample tube to resuspend the exosomes and reduce the disulfide bonds at 60 °C for 30 min. The samples were then alkylated by iodoacetamide in the dark for 30 min, followed by digestion for 16 h using trypsin (Thermo Scientific #90057) at 37 °C. Trifluoroacetic acid was added to the sample tube to a final concentration of 0.1% (v/v) to stop digestion. The samples were then incubated at 37 °C for 40 min to cleavage the detergent. The samples were then centrifuged at 15,000× *g* for 5 min, and the supernatants were transferred into sample vials for LC-MS analysis.

Digested peptide samples were analyzed on an Orbitrap Fusion tribrid mass spectrometer (Thermo Scientific, New York, NY, USA), to which an Ultimate 3000 nano-UPLC system (Thermo Scientific) was connected [24,25]. Furthermore, 2 μL of peptide samples were first trapped on a Pepmap100 C18 peptide trap (5 μm, 0.3 × 5 mm) and then washed at 20 μL/min using 2% acetonitrile with 0.1% formic acid for 10 min [18,19]. Next, the cleaned peptides were washed off the trap and further separated on a Pepman 100 RSLC C18 column (2.0 μm, 75 μm × 150 mm) at 40 °C using a gradient of between 2% and 40% acetonitrile with 0.1% formic acid over 40 min at a flow rate of 300 nL/min [24,25]. LC-MS/MS analysis was performed using data-dependent acquisition in positive mode with the Orbitrap MS analyzer for precursor scans at 120,000 FWHM (full width at half maximum) from 300 to 1500 m/z, and the ion-trap MS analyzer for MS/MS scans at top-speed mode (3 s cycle time) [24,25]. Collision-induced dissociation method was used to fragment the precursor peptides with a normalized energy level of 30%. Raw MS and MS/MS spectrum for each sample were filtered and processed using the Proteome Discoverer software by Thermo Scientific (v1.4) and then submitted to SequestHT search algorithm against the Uniprot human database (10 ppm precursor ion mass tolerance: 10 ppm, product ion mass tolerance: 0.6 Da, static carbamidomethylation of +57.021 Da). The Percolator PSM validator algorithm was used for peptide spectrum matching validation and false discovery rate estimation.

### 2.4. Normalization, Statistical Analysis, and Pathway Analysis of Female-Specific Protein

The peptide spectrum match (PSM) count for each identified protein in the LC-MS/MS search results was used as a semi-quantitative measure for protein expression level. The PSM count for each protein in a specific sample was first normalized using the sum of the PSM counts for all proteins in that sample. Then, the mean PSM count for the three replicates in each group was calculated for each protein and further used for statistical analysis [24,25].

Protein content was compared between the OA vs. non-OA EVs. EdgeR R package was used to perform trimmed mean normalization (TMM), then the difference for protein expression between the groups (OA and non-OA) was analyzed. Proteins up-regulated or down-regulated with a *p*-value cutoff of 0.05 were considered differentially expressed for further analyses. Gene Ontology pathway analyses were conducted using the Database for Annotation, Visualization, and Integrated Discovery (DAVID) [26,27] and QuickGO [28] on differentially expressed protein genes. Uniprot Knowledgebase (UniProtKB) protein descriptions and gene products were imported into DAVID and QuickGO for statistical analyses and GO term annotation based on integrated biological, molecular, and cellular pathways of the differentially expressed proteins.

## 3. Results

### 3.1. EV Protein Cargo Differs Significantly between Male and Female Patients with OA

Previously, we characterized synovial fluid-derived EVs and reported that these EV’s miRNA cargo alters in a gender-specific manner [15]. In this manuscript, we used the same EVs (previously characterized), and that is why we did not show the EV characterization data (please see Kolhe et al. 2017 [15]). In this study, we performed mass spectrometry analysis on synovial fluid-derived EVs proteins (male non-OA, *n* = 7 and OA, *n* = 7 and female non-OA, *n* = 8 and OA, *n* = 10). Previously, we have shown CD81, CD63, and Tsg101 markers on EVs using Western blot and CD9 using an immuno-gold label [15]. For this study, we used the same patient samples for mass spectrometry.

**Figure 1:**
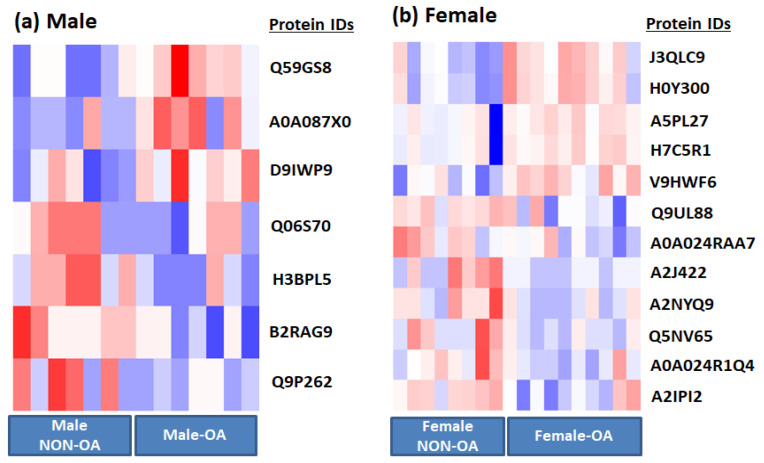
Extracellular vesicle (EV) protein cargo differs in osteoarthritis (OA) synovial fluid. Heat-map of (**a**) male OA (*n* = 7) and non-OA (*n* = 7) and (**b**) female OA (*n* = 10) and non-OA (*n* = 8). Differences between normal and OA patients were examined using Student’s *t*-test, and only those that were significantly different at the *p* value 0.05 level were selected.

**Figure 2:**
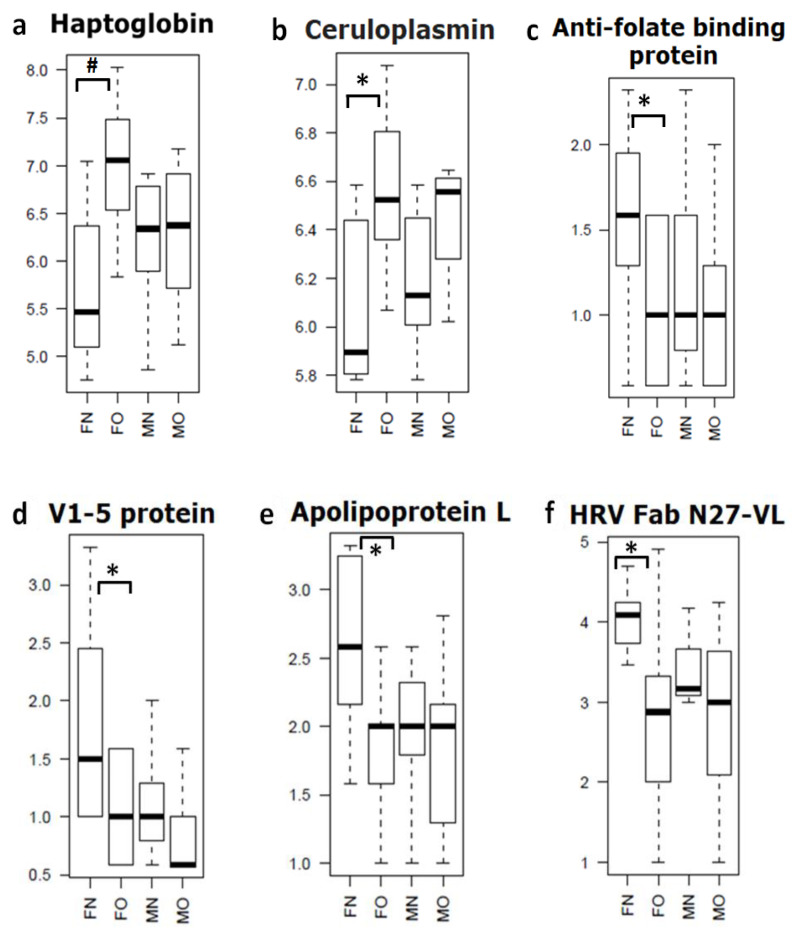
Synovial fluid-derived EV cargo proteins significantly dysregulated in female samples. (**a**) Haptoglobin, (**b**) Ceruloplasmin, (**c**) Anti-folate binding protein, (**d**) V1-5 protein, (**e**) Apolipoprotein L and (**f**) HRV Fab N27-VL. Differences between normal and OA patients were examined using Student’s *t*-test (FN = female non-OA (*n* = 8), FO = female OA (*n* = 10), MN = male non-OA (*n* = 7), MO = male OA (*n* = 7), * *p* = 0.04, **#** *p* = 0.01).

**Figure 3:**
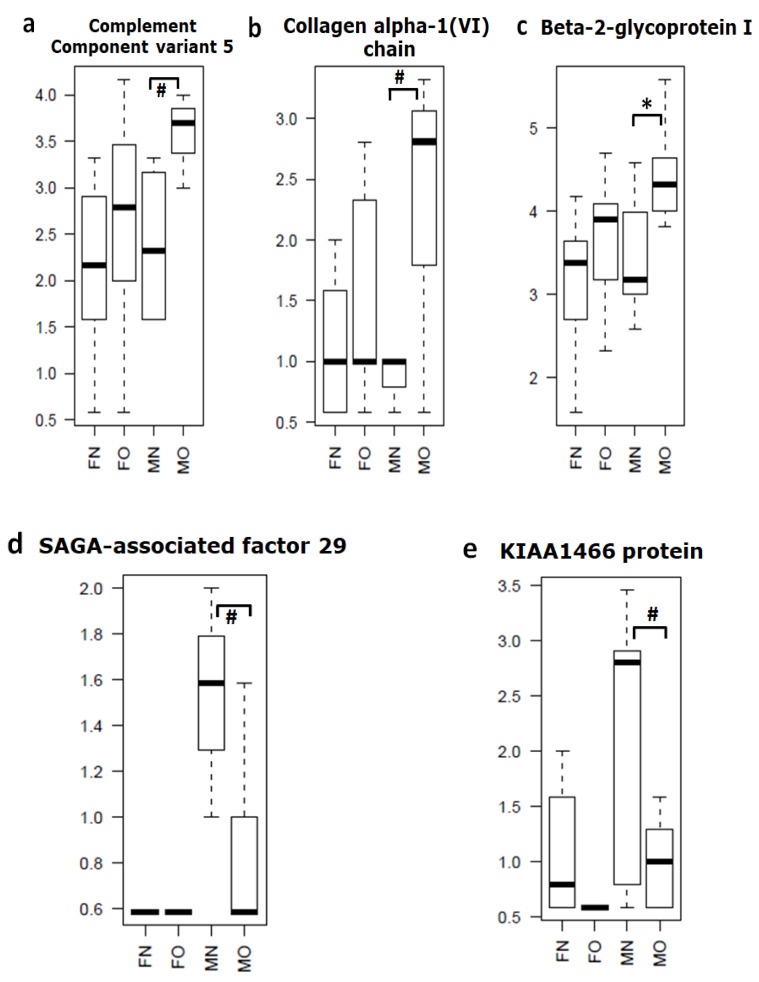
Synovial fluid-derived EV cargo proteins significantly dysregulated in male samples (*n* = 8–10/group). (**a**) Complement Component variant 5, (**b**) Collagen alpha-1 (VI) chain, (**c**) Beta-2-glycoprotein I, (**d**) SAGA-associated factor 29 and (**e**) KIAA1466 protein. Differences between normal and OA patients were examined using Student’s *t*-test (FN = female non-OA (*n* = 8), FO = female OA (*n* = 10), MN = male non-OA (*n* = 7), MO = male OA (*n* = 7), * *p* = 0.04, # *p* = 0.01).

Based on mass spectrometry protein profiling, we identified multiple gender (male and female) specific differential proteins in OA and non-OA exosomes (Table 1 and Figure 1). For example, we found haptoglobin, orosomucoid, and ceruloplasmin were significantly (*p* = 0.01) up-regulated, whereas apolipoprotein was down-regulated (*p* = 0.04) in female OA exosomes (Table 1a and Figure 2). In males, we discovered β-2-glycoprotein and complement component 5 significantly up-regulated (*p* = 0.003), and SAGA-associated factor 29 (*p* = 0.005) down-regulated in male OA exosomes (Table 1b and Figure 3). Our data, therefore, suggest that proteins carried by EVs in the synovial fluid are significantly altered with the osteoarthritic condition and are highly sex-specific.

### 3.2. DAVID and QuickGO Analysis of Differentially Expressed Proteins

To analyze the functions and effects of the differentially expressed proteins on various pathways, Database for Annotation, Visualization, and Integrated Discovery (DAVID) and Quick GO annotation analyses were performed. DAVID annotation analysis revealed extensive involvement of these proteins in endopeptidase and hydrolase activities, vesicle transport and receptor-mediated endocytosis, and a robust, defensive immune response to stress and stimulus in the OA exosomes of synovial fluid in women. Several proteins are involved, and their expression up-regulated several folds in each of the processes, the most enriched being blood microparticle involvement, endocytosis, and endopeptidase and hydrolase activity. The details of the DAVID annotation analysis are shown in the Table 2.

QuickGO annotation analysis revealed extensive involvement of these proteins in multiple biological (lipid transport, regulation of immune system process, lipoprotein metabolic process), molecular (hydrolase activity, ATP-dependent helicase activity, nucleic acid binding), and cellular pathways (extracellular region, nucleus, nucleoplasm) in OA exosomes. Furthermore, these proteins were shown to be primarily involved in regulating the immune system process in women with OA (Table 3). We also performed DAVID and QuickGO analysis on male EVs cargo proteins but did not find significant changes (Supplementary Tables S1 and S2).

## 4. Discussion

The synovial fluid consists of secretory products from synovium, cartilage, and other components of articular joints. Several recent studies have analyzed the synovial fluid for miRNAs, cytokines, and proteins to better understand the pathophysiological status of OA [13,14,15,29]. Recently, we reported that synovial fluid-derived EVs carry specific miRNAs in osteoarthritis patients, and importantly, these miRNAs were gender-specific. EVs are 40–100 nm in diameter and contain various components, including proteins, lipids, and miRNA [16]. Previously, we also characterized the synovial fluid-derived EVs of OA and non-OA patients [15]. We found that EVs isolated from the synovial fluid are round-shaped vesicles, ~100 (±10) nm diameter size ranges, with no change in concentration between OA and non-OA patients [15]. We also found expression of CD9 (using electron microscopy immuno-gold staining) and Tsg101, CD63, and CD81 (using Western blot), which are the markers for EVs [15].

It has been previously reported that EV protein plays a vital role in various age-related and degenerative diseases [30,31,32,33]. Considering the critical role of EV protein cargoes in the progression of various age-related diseases, it is clinically relevant to analyze EVs’ protein content of OA synovial fluid. We hypothesized that synovial fluid-derived EV protein content not only differs in OA condition, compared to non-OA, but also differs in a sex-specific manner. We analyzed the composition of EV protein cargo using mass spectrophotometry in the synovial fluid of males and females with OA compared with the controls.

Our mass spectrophotometry data showed that numbers of proteins were differentially present in the EV cargo of OA samples. Interestingly, as expected, female EV protein profiles were completely different to males’. In females, we found that haptoglobin, orosomucoid, and ceruloplasmin, were significantly up-regulated and apolipoprotein L down-regulated. In contrast, in males, β-2-glycoprotein and complement component 5 proteins were up-regulated and SAGA-associated factor 29 significantly down-regulated in OA EVs. To the best of our knowledge, our study is the first to characterize EV protein cargo from the synovial fluid of non-OA and OA patients in a sex-specific manner.

Our data indicate that these different EV protein cargoes can help to develop gender-specific biomarkers for diagnosis and understanding of OA pathophysiology. For example, the haptoglobin, which is elevated in female synovial fluid EV cargo, is known to be dysregulated in several other female-related pathological conditions [34,35,36,37]. Haptoglobin is increased during periods of inflammation and has been identified as an acute-phase glycoprotein [38]. Haptoglobin has been shown to have increased production during inflammation via IL-6 and TNF-alpha and serves as a modulator of inflammation [38,39]. Haptoglobin fragments are detectable in OA serum, indicating an alteration in protein pattern during OA [40,41]. Haptoglobin has also been shown to correlate with OA severity, with increased levels in the synovial fluid of patients with more symptomatic OA [42]. Elevated levels of haptoglobin correlate with an increased inflammatory state of cytokines, and cell-mediated inflammation [34,35,36,37].

Another important acute-phase protein, orosomucoid (also known as AGP-1), only up-regulated significantly in female synovial fluid-derived EVs cargo. This protein has been dysregulated in numerous disease processes, including liver disease, cancer, HIV, and other inflammatory conditions [43,44,45]. Orosomucoid has also been shown to interact with TLR-4 and CD14 and modulate immune response [45,46,47]. Another study has shown that orosomucoid is down-regulated in the presence of the female hormone estrogen via an estrogen receptor-dependent pathway [48]. This indicates that these proteins play an important role in gender-specific immunomodulation of synovial health. It is possible that differential EV protein cargo in female synovial fluid may be due to a decline in estrogen levels. It is well known that declining estrogen levels are inversely related to increased incidence and severity of OA in females [49,50,51,52]. We previously demonstrated that estrogen inhibitor treatment alters miRNA cargo in female synovial fibroblast cells [15]. Surprisingly, we did not find significant changes in pro-inflammatory cytokines (such as IL-1, IL-6, and TNF-a), which play an important role in OA progression.

**Figure 4:**
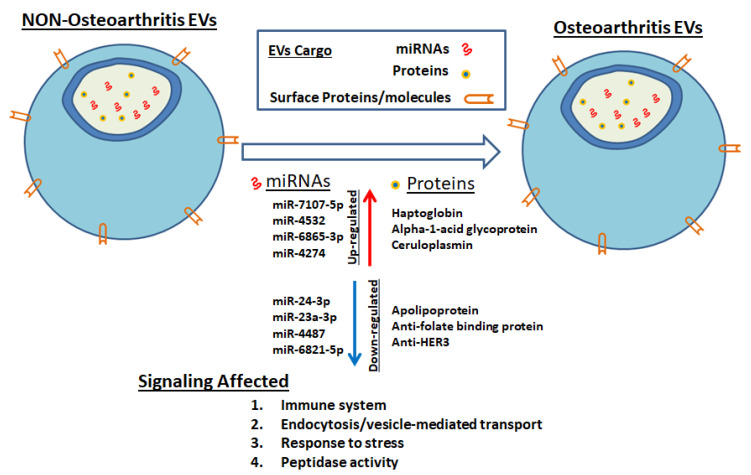
Schematic diagram showing the involvement of synovial fluid-derived EV cargo (microRNAs and proteins) in signaling pathways.

To further investigate the role of these proteins in signaling pathways, we performed bioinformatics analysis using DAVID and QuickGO analysis on these differentially regulated proteins. The female EV protein cargo demonstrated a complex web of pathway connections in which these proteins interact to elicit several cellular, molecular, and biological processes. These proteins are involved in endopeptidase and hydrolase activity, immune receptor-mediated endocytosis, receptor-mediated phagocytosis, immune system regulation, and response to stress and activation. Previously, we reported that female synovial fluid-derived EV miRNA cargo also affects similar signaling pathways [15]; specifically, the immune system and response to stress signaling pathways. MicroRNA cargo from our previous study [15] and protein EV cargo from this study showed strong evidence that the immune system plays a massive role in the development of OA in females (Figure 4). These findings indicate a complex interplay of dysregulated synovial fluid-derived EV cargos (miRNAs and proteins) in female OA.

Previous studies have shown the significance of EVs in the pathogenesis of OA and their potential to use them as markers for disease severity and progression [18,53,54]. Our data strongly agrees with published literature but also suggests that female and male OA should be investigated separately to get more accurate and valuable information. Our data indicate that EV-derived protein plays a vital role in female OA and its pathogenesis, helping to explain the increased prevalence and severity of OA in women. Further in-depth in vitro and in vivo studies should be investigated, as prevention, screening, and treatment can be personalized based on gender for more clinical successful treatment of OA.

Our study does have limitations, the most important being that we had a small sample size. Larger sample sizes are needed, as well as various stages of OA in women and men should be investigated. To conclude, ours is the first study to show male and female-specific protein EV profiling in OA. Exosomes and their contents are vital to understanding the pathophysiology of OA in a gender-specific manner. These proteins contribute to several female-specific biologic, molecular, and cellular pathways and may help to explain the increased prevalence and severity of OA in women.

## Figures and Tables

**Table 1:** Showing a list of synovial fluid-derived EV cargo proteins in (**a**) female and (**b**) male in OA compared to non-OA.

Serial.No	Gene Name/Description	Fold Change	***p* Value**
(**a**)			
1	Haptoglobin (Fragment) GN = HP	2.46	0.001
2	Haptoglobin GN = HP	2.32	0.001
3	V1-5 protein (Fragment) GN = V1-5	0.60	0.014
4	Alpha-1-acid glycoprotein GN = HEL-S-153w	1.47	0.022
5	Anti-HER3 scFv (Fragment)	0.65	0.024
6	Apolipoprotein L, 1, isoform CRA_c GN = APOL1	0.55	0.024
7	Anti-folate binding protein (Fragment) GN = HuVH8B	0.63	0.027
8	Complement component 1, q subcomponent, C chain, isoform CRA_a GN = C1QC	0.69	0.028
9	HRV Fab N27-VL (Fragment)	0.45	0.032
10	Ceruloplasmin (Fragment) GN = CP	1.93	0.032
11	CP protein GN = CP	2.01	0.034
12	Myosin-reactive immunoglobulin heavy chain variable region (Fragment)	0.80	0.038
(**b**)			
1	SAGA-associated factor 29 homolog GN = CCDC101	0.62	0.0002
2	Collagen alpha-1(VI) chain GN = COL6A1	2.36	0.0060
3	Complement component 5 variant (Fragment)	2.77	0.0072
4	KIAA1466 protein (Fragment) GN = KIAA1466	0.48	0.0085
5	Beta-2-glycoprotein I (Fragment)	1.94	0.0215
6	cDNA, FLJ94908, highly similar to *Homo sapiens* PPAR binding protein (PPARBP), mRNA	0.49	0.0283
7	3’-5’ exonuclease TREX2 long form	0.72	0.0320

**Table 2:** Selected Database for Annotation, Visualization, and Integrated Discovery (DAVID) Gene Ontology (GO) pathways affected by EVs cargo proteins in female OA.

GO Term	*p* Value
GO:0006898~receptor-mediated endocytosis	0.00072
GO:0004252~serine-type endopeptidase activity	0.00134
GO:0008236~serine-type peptidase activity	0.00163
GO:0017171~serine hydrolase activity	0.00167
GO:0006898~receptor-mediated endocytosis	0.00232
GO:0005576~extracellular region	0.00257
GO:0004175~endopeptidase activity	0.00495
GO:0006897~endocytosis	0.00891
GO:0070011~peptidase activity, acting on L-amino acid peptides	0.00996
GO:0008233~peptidase activity	0.01066
GO:0002376~immune system process	0.01125
GO:0016787~hydrolase activity	0.01296
GO:0016192~vesicle-mediated transport	0.04203
GO:0006955~immune response	0.04508
GO:0005576~extracellular region	0.05286
GO:0072562~blood microparticle	0.00041
GO:0005576~extracellular region	0.00257
GO:0002376~immune system process	0.01125
GO:0005615~extracellular space	0.02962
GO:0006950~response to stress	0.03508
GO:0006952~defense response	0.04330

**Table 3:** Selected QuickGO pathways affected by EVs cargo proteins in female OA.

Biological Function Involved	Signaling Involved
biological_process	lipid transport
biological_process	regulation of immune system process
biological_process	lipoprotein metabolic process
biological_process	receptor-mediated endocytosis
biological_process	Fc-epsilon receptor signaling pathway
biological_process	Fc-gamma receptor signaling pathway involved in phagocytosis
biological_process	complement activation
biological_process	complement activation, classical pathway
biological_process	proteolysis
biological_process	leukocyte migration
biological_process	regulation of immune response
biological_process	DNA recombination
biological_process	regulation of complement activation
molecular_function	hydrolase activity
molecular_function	ATP-dependent helicase activity
molecular_function	nucleic acid binding
molecular_function	serine-type endopeptidase activity
molecular_function	helicase activity
molecular_function	nucleotide binding
molecular_function	lipid binding
molecular_function	ATP binding
molecular_function	hemoglobin binding
cellular_component	extracellular region
cellular_component	nucleus
cellular_component	nucleoplasm
cellular_component	collagen trimer
cellular_component	extracellular space

## Supplementary Materials

The following are available online at https://www.mdpi.com/2075-1729/10/12/337/s1, Table S1. Selected David Gene Ontology pathways affected by EVs cargo proteins in male OA. Table S2. Selected QuickGO pathways affected by EVs cargo proteins in male OA.

## References

[1] Murphy L., Helmick C.G. (2012). The impact of osteoarthritis in the United States: A population-health perspective. Am. J. Nurs..

[2] Lawrence R.C., Felson D.T., Helmick C.G., Arnold L.M., Choi H., Deyo R.A., Gabriel S., Hirsch R., Hochberg M.C., Hunder G.G. (2008). Estimates of the prevalence of arthritis and other rheumatic conditions in the United States: Part II. Arthritis Rheum..

[3] Hame S.L., Alexander R.A. (2013). Knee osteoarthritis in women. Curr. Rev. Musculoskelet. Med..

[4] O’connor M.I. (2007). Sex Differences in Osteoarthritis of the Hip and Knee. J. Am. Acad. Orthop. Surg..

[5] Piva S.R., Susko A.M., Khoja S.S., Josbeno D.A., Fitzgerald G.K., Toledo F.G.S. (2015). Links between osteoarthritis and diabetes: Implications for management from a physical activity perspective. Clin. Geriatr. Med..

[6] Menashe L., Hirko K., Losina E., Kloppenburg M., Zhang W., Li L., Hunter D.J. (2012). The diagnostic performance of MRI in osteoarthritis: A systematic review and meta-analysis. Osteoarthr. Cartil..

[7] Iolascon G., Gimigliano F., Moretti A., De Sire A., Migliore A., Brandi M., Piscitelli P. (2017). Early osteoarthritis: How to define, diagnose, and manage. A systematic review. Eur. Geriatr. Med..

[8] De Sire A., De Sire R., Petito V., Masi L., Cisari C., Gasbarrini A., Scaldaferri F., Invernizzi M. (2020). Gut–Joint Axis: The Role of Physical Exercise on Gut Microbiota Modulation in Older People with Osteoarthritis. Nutrients.

[9] Damiani C., Mangone M., Paoloni M., Goffredo M., Franceschini M., Servidio M., Pournajaf S., Santilli V., Agostini F., Bernetti A. (2020). Trade-Offs with rehabilitation Effectiveness (REs) and Efficiency (REy) in a sample of Italian disabled persons in a in post-acuity rehabilitation unit. Ann. Ig..

[10] De Sire A., Stagno D., Minetto M.A., Cisari C., Baricich A., Invernizzi M. (2020). Long-term effects of intra-articular oxygen-ozone therapy versus hyaluronic acid in older people affected by knee osteoarthritis: A randomized single-blind extension study. J. Back Musculoskelet Rehabil..

[11] Santilli V., Mangone M., Paoloni M., Agostini F., Alviti F., Bernetti A. (2018). Comment on Early Efficacy of Intra-Articular HYADD^®^ 4 (Hymovis^®^) Injections for Symptomatic Knee Osteoarthritis. Joints.

[12] Rabini A., De Sire A., Marzetti E., Gimigliano R., Ferriero G., Piazzini D.B., Iolascon G., Gimigliano F. (2015). Effects of focal muscle vibration on physical functioning in patients with knee osteoarthritis: A randomized controlled trial. Eur. J. Phys. Rehabil. Med..

[13] Liao W., Li Z., Zhang H., Li J., Wang K., Yang Y. (2015). Proteomic analysis of synovial fluid as an analytical tool to detect candidate biomarkers for knee osteoarthritis. Int. J. Clin. Exp. Pathol..

[14] Mabey T., Honsawek S., Tanavalee A., Yuktanandana P., Wilairatana V., Poovorawan Y. (2016). Plasma and synovial fluid inflammatory cytokine profiles in primary knee osteoarthritis. Biomarkers.

[15] Kolhe R., Hunter M., Liu S., Jadeja R.N., Pundkar C., Mondal A.K., Mendhe B., Drewry M., Rojiani M.V., Liu Y. (2017). Gender-specific differential expression of exosomal miRNA in synovial fluid of patients with osteoarthritis. Sci. Rep..

[16] Rashed M.H., Bayraktar E., Helal G.K., Abd-Ellah M.F., Amero P., Chavez-Reyes A., Rodriguez-Aguayo C. (2017). Exosomes: From Garbage Bins to Promising Therapeutic Targets. Int. J. Mol. Sci..

[17] Withrow J., Murphy C., Liu Y., Hunter M., Fulzele S., Hamrick M.W. (2016). Extracellular vesicles in the pathogenesis of rheumatoid arthritis and osteoarthritis. Arthritis Res..

[18] Domenis R., Zanutel R., Caponnetto F., Toffoletto B., Cifù A., Pistis C., Di Benedetto P., Causero A., Pozzi M., Bassini F. (2017). Characterization of the pro-inflammatory profile of synovial fluid-derived exosomes of patients with osteoarthritis. Mediat. Inflamm..

[19] Berckmans R.J., Nieuwland R., Kraan M.C., Schaap M.C.L., Pots D., Smeets T.J.M., Sturk A., Tak P.P. (2005). Synovial microparticles from arthritic patients modulate chemokine and cytokine release by synoviocytes. Arthritis Res. Ther..

[20] Wang Y., Yu D., Liu Z., Zhou F., Dai J., Wu B., Zhou J., Heng B.C., Zou X.H., Ouyang H. (2017). Exosomes from embryonic mesenchymal stem cells alleviate osteoarthritis through balancing synthesis and degradation of cartilage extracellular matrix. Stem Cell Res. Ther..

[21] Tofiño-Vian M., Guillén M.I., Del Caz M.D.P., Silvestre A., Alcaraz M.J. (2018). Microvesicles from Human Adipose Tissue-Derived Mesenchymal Stem Cells as a New Protective Strategy in Osteoarthritic Chondrocytes. Cell. Physiol. Biochem..

[22] Helwa I., Cai J., Drewry M.D., Zimmerman A., Dinkins M.B., Khaled M.L., Seremwe M., Dismuke W.M., Bieberich E., Stamer W.D. (2017). A Comparative Study of Serum Exosome Isolation Using Differential Ultracentrifugation and Three Commercial Reagents. PLoS ONE.

[23] Rider M.A., Hurwitz S.N., Meckes D.G. (2016). ExtraPEG: A Polyethylene Glycol-Based Method for Enrichment of Extracellular Vesicles. Sci. Rep..

[24] Sharma S., Bollinger K.E., Kodeboyina S.K., Zhi W., Patton J., Bai S., Edwards B., Ulrich L., Bogorad D., Sharma A. (2018). Proteomic Alterations in Aqueous Humor From Patients With Primary Open Angle Glaucoma. Investig. Opthalmol. Vis. Sci..

[25] Dasari R., Zhi W., Bonsack F., Sukumari-Ramesh S. (2020). A Combined Proteomics and Bioinformatics Approach Reveals Novel Signaling Pathways and Molecular Targets After Intracerebral Hemorrhage. J. Mol. Neurosci..

[26] Huang D.W., Sherman B.T., Lempicki R.A. (2009). Systematic and integrative analysis of large gene lists using DAVID bioinformatics resources. Nat. Protoc..

[27] Huang D.W., Sherman B.T., Lempicki R.A. (2009). Bioinformatics enrichment tools: Paths toward the comprehensive functional analysis of large gene lists. Nucleic Acids Res..

[28] Binns D., Dimmer E., Huntley R., Barrell D., O’Donovan C., Apweiler R. (2009). QuickGO: A web-based tool for Gene Ontology searching. Bioinformatics.

[29] Munjal A., Bapat S., Hubbard D., Hunter M., Kolhe R., Fulzele S. (2019). Advances in Molecular biomarker for early diagnosis of Osteoarthritis. Biomol. Concepts.

[30] Kruger S., Elmageed Z.Y.A., Hawke D.H., Wörner P.M., Jansen D.A., Abdel-Mageed A.B., Alt E., Izadpanah R. (2014). Molecular characterization of exosome-like vesicles from breast cancer cells. BMC Cancer.

[31] Eitan E., Green J., Bodogai M., Mode N.A., Bæk R., Jørgensen M.M., Freeman D.W., Witwer K.W., Zonderman A.B., Biragyn A. (2017). Age-Related Changes in Plasma Extracellular Vesicle Characteristics and Internalization by Leukocytes. Sci. Rep..

[32] Fulzele S., Mendhe B., Khayrullin A., Johnson M., Kaiser H., Liu Y., Isales C.M., Hamrick M.W. (2019). Muscle-derived miR-34a increases with age in circulating extracellular vesicles and induces senescence of bone marrow stem cells. Aging.

[33] Saha B., Momen-Heravi F., Furi I., Kodys K., Catalano D., Gangopadhyay A., Haraszti R., Satishchandran A., Iracheta-Vellve A., Adejumo A. (2018). Extracellular vesicles from mice with alcoholic liver disease carry a distinct protein cargo and induce macrophage activation through heat shock protein 90. Hepatology.

[34] Sammour R.N., Nakhoul F., Levy A.P., Miller-Lotan R., Nakhoul N., Awad H.R., Gonen R., Ohel G. (2010). Haptoglobin phenotype in women with preeclampsia. Endocrine.

[35] Álvarez-Blasco F., Martínez-García M.Á., Luque-Ramírez M., Parraza N., Millán J.L.S., Escobar-Morreale H.F. (2009). Role of Haptoglobin in Polycystic Ovary Syndrome (PCOS), Obesity and Disorders of Glucose Tolerance in Premenopausal Women. PLoS ONE.

[36] Berkova N., Lemay A., Dresser D.W., Fontaine J.-Y., Kerizit J., Goupil S. (2001). Haptoglobin is present in human endometrium and shows elevated levels in the decidua during pregnancy. Mol. Hum. Reprod..

[37] Tang K.Y., Huang S.-Y., Cheng T.-M., Bai C.-H., Chang J.-S. (2020). Haptoglobin phenotype influences the effectiveness of diet-induced weight loss in middle-age abdominally obese women with metabolic abnormalities. Clin. Nutr..

[38] Huntoon K.M., Wang Y., Eppolito C.A., Barbour K.W., Berger F.G., Shrikant P.A., Kawasaki T. (2008). The acute phase protein haptoglobin regulates host immunity. J. Leukoc. Biol..

[39] Smeets M.B., Fontijn J., Kavelaars A., Pasterkamp G., De Kleijn D.P. (2003). The acute phase protein haptoglobin is locally expressed in arthritic and oncological tissues. Int. J. Exp. Pathol..

[40] Ghafouri B., Carlsson A., Holmberg S., Thelin A., Tagesson C. (2016). Biomarkers of systemic inflammation in farmers with musculoskeletal disorders; a plasma proteomic study. BMC Musculoskelet. Disord..

[41] Fernández-Puente P., Calamia V., González-Rodríguez L., Lourido L., Camacho-Encina M., Oreiro N., Ruiz-Romero C., Blanco F.J. (2017). Multiplexed mass spectrometry monitoring of biomarker candidates for osteoarthritis. J. Proteom..

[42] Park H.J., Oh M.-K., Kim N.-H., Cho M.-L., Kim I.-S. (2013). Identification of a specific haptoglobin C-terminal fragment in arthritic synovial fluid and its effect on interleukin-6 expression. Immunology.

[43] Magid E., Guldager H., Hesse D., Christiansen M.S. (2005). Monitoring Urinary Orosomucoid in Acute Inflammation: Observations on Urinary Excretion of Orosomucoid, Albumin, α1-Microglobulin, and IgG. Clin. Chem..

[44] Ligresti G., Aplin A.C., Dunn B.E., Morishita A., Nicosia R.F. (2012). The Acute Phase Reactant Orosomucoid-1 Is a Bimodal Regulator of Angiogenesis with Time- and Context-Dependent Inhibitory and Stimulatory Properties. PLoS ONE.

[45] Luo Z., Lei H., Sun Y., Liu X., Su D.-F. (2015). Orosomucoid, an acute response protein with multiple modulating activities. J. Physiol. Biochem..

[46] Rangé H., Poitou C., Boillot A., Ciangura C., Katsahian S., Lacorte J.-M., Czernichow S., Meilhac O., Bouchard P., Chaussain C. (2013). Orosomucoid, a New Biomarker in the Association between Obesity and Periodontitis. PLoS ONE.

[47] Komori H., Watanabe H., Shuto T., Kodama A., Maeda H., Watanabe K., Kai H., Otagiri M., Maruyama T. (2012). α1-Acid Glycoprotein Up-regulates CD163 via TLR4/CD14 Protein Pathway. J. Biol. Chem..

[48] Sun Y., Qin Z., Wan J.-J., Wang P.-Y., Yang Y.-L., Yu J.-G., Hu B.-H., Su D.-F., Luo Z.-M., Liu X. (2018). Estrogen weakens muscle endurance via estrogen receptor-p38 MAPK-mediated orosomucoid (ORM) suppression. Exp. Mol. Med..

[49] Roman-Blas J., Castañeda S., Largo R., Herrero-Beaumont G. (2009). Osteoarthritis associated with estrogen deficiency. Arthritis Res. Ther..

[50] Jung J.H., Bang C.H., Song G.G., Kim C., Kim J.-H., Choi S.J. (2019). Knee osteoarthritis and menopausal hormone therapy in postmenopausal women. Menopause.

[51] Watt F.M. (2018). Musculoskeletal pain and menopause. Post Reprod. Health.

[52] Khadilkar S.S. (2019). Musculoskeletal Disorders and Menopause. J. Obstet. Gynecol. India.

[53] Zhao Y., Xu J. (2018). Synovial fluid-derived exosomal lncRNA PCGEM1 as biomarker for the different stages of osteoarthritis. Int. Orthop..

[54] Kato T., Miyaki S., Ishitobi H., Nakamura Y., Nakasa T., Lotz M.K., Ochi M. (2014). Exosomes from IL-1β stimulated synovial fibroblasts induce osteoarthritic changes in articular chondrocytes. Arthritis Res. Ther..

