# Supplementary material for: Sex-Specific Differences in Extracellular Vesicle Protein Cargo in Synovial Fluid of Patients with Osteoarthritis
Source: Life (Basel). 2020 Dec 10;10(12):337. doi: 10.3390/life10120337 (PMC7763294; doi:10.3390/life10120337)
Supplement: Supplementary file 1 [file life-10-00337-s001.pdf]

life

MDPI

Supplementary Materials

Sex-specific differences in Extracellular vesicle protein cargo in synovial fluid of patients with osteoarthritis.

Table S1. Selected David Gene Ontology pathways affected by EVs cargo proteins in male OA.

GO	Term	P-value
GO:0032101	regulation of response to external stimulus	0.065925
GO:0048646	anatomical structure formation involved in morphogenesis	0.065925
GO:0044093	positive regulation of molecular function	0.070259
GO:0051240	positive regulation of multicellular organismal process	0.071287
GO:0006950	response to stress	0.081534
GO:0032991	protein-containing complex	0.065925
GO:0070062	extracellular exosome	0.083457
GO:0043230	extracellular organelle	0.088329
GO:1903561	extracellular vesicle	0.088329
GO:0005615	extracellular space	0.155983
GO:0098772	molecular function regulator	0.165078
GO:0005515	protein binding	0.170425
GO:0005488	binding	0.32984
GO:0003674	molecular_function	0.537787

Table S2. Selected QuickGO pathways affected by EVs cargo proteins in male OA.

Biological Function involved	Signaling Involved
biological_process	regulation of response to external stimulus
biological_process	anatomical structure formation involved in morphogenesis
biological_process	positive regulation of molecular function
biological_process	positive regulation of multicellular organismal process
biological_process	response to stress
biological_process	positive regulation of response to stimulus
biological_process	anatomical structure morphogenesis
biological_process	response to external stimulus
biological_process	regulation of molecular function
biological_process	macromolecule localization
biological_process	cellular response to chemical stimulus
biological_process	protein metabolic process
biological_process	regulation of multicellular organismal process
molecular_function	molecular function regulator
molecular_function	protein binding
molecular_function	binding
molecular_function	molecular_function
molecular_function	retinoic acid receptor binding
molecular_function	thyroid hormone receptor coactivator activity
molecular_function	exodeoxyribonuclease activity
molecular_function	3'-5'-exodeoxyribonuclease activity
molecular_function	exodeoxyribonuclease activity, producing 5'-phosphomonoesters

cellular_component	protein-containing complex
cellular_component	extracellular exosome
cellular_component	extracellular organelle
cellular_component	extracellular vesicle
cellular_component	extracellular space
cellular_component	membrane-bounded organelle
cellular_component	membrane-enclosed lumen
cellular_component	organelle lumen
cellular_component	intracellular organelle lumen
cellular_component	vesicle
cellular_component	organelle
cellular_component	extracellular region
cellular_component	intracellular organelle
cellular_component	nucleus
